# Development of Personas and Journey Maps for Artificial Intelligence Agents Supporting the Use of Health Big Data: Human-Centered Design Approach

**DOI:** 10.2196/67272

**Published:** 2025-01-08

**Authors:** Yoon Heui Lee, Hanna Choi, Soo-Kyoung Lee

**Affiliations:** 1 Department of Nursing, Graduate School Keimyung University Daegu Republic of Korea; 2 Department of Nursing Science Nambu University Gwangju Republic of Korea; 3 Big Data Convergence and Open Sharing System Seoul National University Seoul Republic of Korea

**Keywords:** analysis, health big data, human-centered design, persona, user journey map, artificial intelligence, human-AI, interviews, users’ experiences

## Abstract

**Background:**

The rapid proliferation of artificial intelligence (AI) requires new approaches for human-AI interfaces that are different from classic human-computer interfaces. In developing a system that is conducive to the analysis and use of health big data (HBD), reflecting the empirical characteristics of users who have performed HBD analysis is the most crucial aspect to consider. Recently, human-centered design methodology, a field of user-centered design, has been expanded and is used not only to develop types of products but also technologies and services.

**Objective:**

This study was conducted to integrate and analyze users’ experiences along the HBD analysis journey using the human-centered design methodology and reflect them in the development of AI agents that support future HBD analysis. This research aims to help accelerate the development of novel human-AI interfaces for AI agents that support the analysis and use of HBD, which will be urgently needed in the near future.

**Methods:**

Using human-centered design methodology, we collected data through shadowing and in-depth interviews with 16 people with experience in analyzing and using HBD. We identified users’ empirical characteristics, emotions, pain points, and needs related to HBD analysis and use and created personas and journey maps.

**Results:**

The general characteristics of participants (n=16) were as follows: the majority were in their 40s (n=6, 38%) and held a PhD degree (n=10, 63%). Professors (n=7, 44%) and health care personnel (n=10, 63%) represented the largest professional groups. Participants’ experiences with big data analysis varied, with 25% (n=4) being beginners and 38% (n=6) having extensive experience. Common analysis methods included statistical analysis (n=7, 44%) and data mining (n=6, 38%). Qualitative findings from shadowing and in-depth interviews revealed key challenges: lack of knowledge on using analytical solutions, crisis management difficulties during errors, and inadequate understanding of health care data and clinical decision-making, especially among non–health care professionals. Three types of personas and journey maps—health care professionals as big data analysis beginners, health care professionals who have experience in big data analytics, and non–health care professionals who are experts in big data analytics—were derived. They showed a need for personalized platforms tailored to the user level, appropriate direction through a navigation function, a crisis management support system, communication and sharing among users, and expert linkage service.

**Conclusions:**

The knowledge obtained from this study can be leveraged in designing an AI agent to support future HBD analysis and use. This is expected to further increase the usability of HBD by helping users perform effective use of HBD more easily.

## Introduction

Since COVID-19, the new normal era has brought innovative advances in digital technology, resulting in various changes in all areas of our lives. Health care is rapidly emerging as one of the most representative and top-priority areas of advanced digital technology [1-3]. Digital health is a generic term for health care–related fields that combine digital technology and medical care, including telehealth, telemedicine, mobile health, wearable and internet of things–based health care, and big data and artificial intelligence (AI)–based health care [4-6]. The purpose of digital health is to create specialized services for consumers based on data- and evidence-based judgment, and its goal is to realize precision health care [3,7,8] through sustainable digital transformation of health care [9].

Precision health care is a new health care paradigm that involves analyzing various information such as genetic and environmental factors, disease history, lifestyle, and individual health information to prevent diseases (preventive health care), maximize treatment effects, and minimize treatment side effects (personalized health care); this also involves the participation of patients in the decision-making process (participatory health care) [10-13]. Having an accurate prediction based on various health data is important in realizing such precision health care [14]. Health care is one of the fastest-growing sectors worldwide and requires an efficient and continuous digital transformation [15]. The digital transformation of health care should consider technological, political, social, cultural, and economic aspects and is a key and strategic field for improving health care quality and creating new value [16].

According to IBM, an individual generates more than 1100 terabytes of health data in their lifetime [17], and this huge amount of data that was previously just discarded can now be shared, transmitted, and stored with advances in the cloud, AI, and social media. Integrating and analyzing such health big data (HBD) can derive new insights for predicting, preventing, and treating diseases [18,19]. Korea has a huge amount of HBD; hence, the development of various government-level policy support and related technologies continues [20,21] and has favorable conditions for the use of HBD. However, the usability of HBD is low. The use of big data is mainly hindered by the absence of experts related to big data analysis, inconsistent data quality, and difficulty in analysis procedures [22,23].

Currently, there are only a few professionals who can manage and analyze big data in Korea. As interest in big data expands, analytic expert training courses are continuously being opened; however, they are still far from enough to meet demands [24]. In addition, considering future demands where the use and influence of big data will further increase, the workforce imbalance is also expected to increase [22,25]. Therefore, training more people with universal capabilities based on an understanding of data can be an effective alternative to meet insufficient workforce demand and increase the use of big data [26]. To this end, a system that supports the overall process of big data analysis is needed so that many people can access HBD more easily for analysis and use. Recently, human-centered design methodology, a field of user-centered design, has been expanded and used not only to develop types of products but also technologies or services [27,28]. This helps in specifically identifying the problem based on the user’s experience, identifying the user’s needs, and reflecting them in the human-centered design [29,30].

AI applications are becoming increasingly common in almost all areas of human life. In addition, the medical field is also accelerating to reflect AI. The rapid proliferation of AI requires new approaches for human-AI interfaces that are different from classic human-computer interfaces [31]. Persona and journey mapping techniques have been used in the literature and industry to elicit requirements for system development. Most of the software developers, users, and managers consider that the journey map and persona techniques are effective in helping understand the software requirements to be developed by the development teams [32].

In developing a system that supports the analysis and use of HBD, reflecting the empirical characteristics of users who actually perform big data analysis is the most important procedure to consider. Therefore, this study aims to understand the empirical characteristics and feelings of users related to big data analysis by building persona and user journey map included in the human-centered design methodology and to understand users’ needs for systems that support HBD analysis and use. This can be used as a design concept for AI agents to support future HBD analysis and use.

Furthermore, this study aims to design an AI agent to support future HBD analysis and use by integrating and analyzing users’ experiences along the HBD analysis and use journey and applying the human-centered design methodology as follows: (1) the persona type is derived by investigating the user’s experience focusing on the HBD analysis and use journey and (2) a customer journey map is created according to the derived persona to identify the needs of users presented by type.

## Methods

### Research Design

This is a methodological study that synthesizes and analyzes users’ experiences according to the big data analysis and use journey; generally, the human-centered design methodology is used before developing an AI agent that supports the analysis and use of HBD.

### Participants

Research participants were secured through convenience and snowball sampling methods for adults aged 19 years or older living in Korea who have experience in big data analysis using HBD for research or work purposes or have experience using the results of the big data analysis. Participants for this study were recruited voluntarily by sending a recruitment notice to individuals identified as meeting the study’s objectives from among those who had participated in a prior demand survey on the analysis and use of HBD [33]. This survey served as a preliminary study for this research, and the individuals identified through it were selected as the primary participants. Additional participants were then recruited through referrals from these primary participants. To ensure diversity within the participant pool, individuals were classified into 4 categories based on their level of analysis experience (beginner or proficient) and their occupational field (health and medical fields or other fields). Efforts were made to select participants evenly across these categories. There were 16 participants in this study. The number of participants was evaluated based on previous research that suggested that a saturation point without new discoveries could be reached when interviewing 6 to 12 participants, and recruitment of participants was terminated at the point when new topics were no longer derived [34]. Data were collected until no new topics emerged from the interviews, and the recruitment of new participants was stopped at the time of saturation [35].

### Procedures

**Figure 1:**
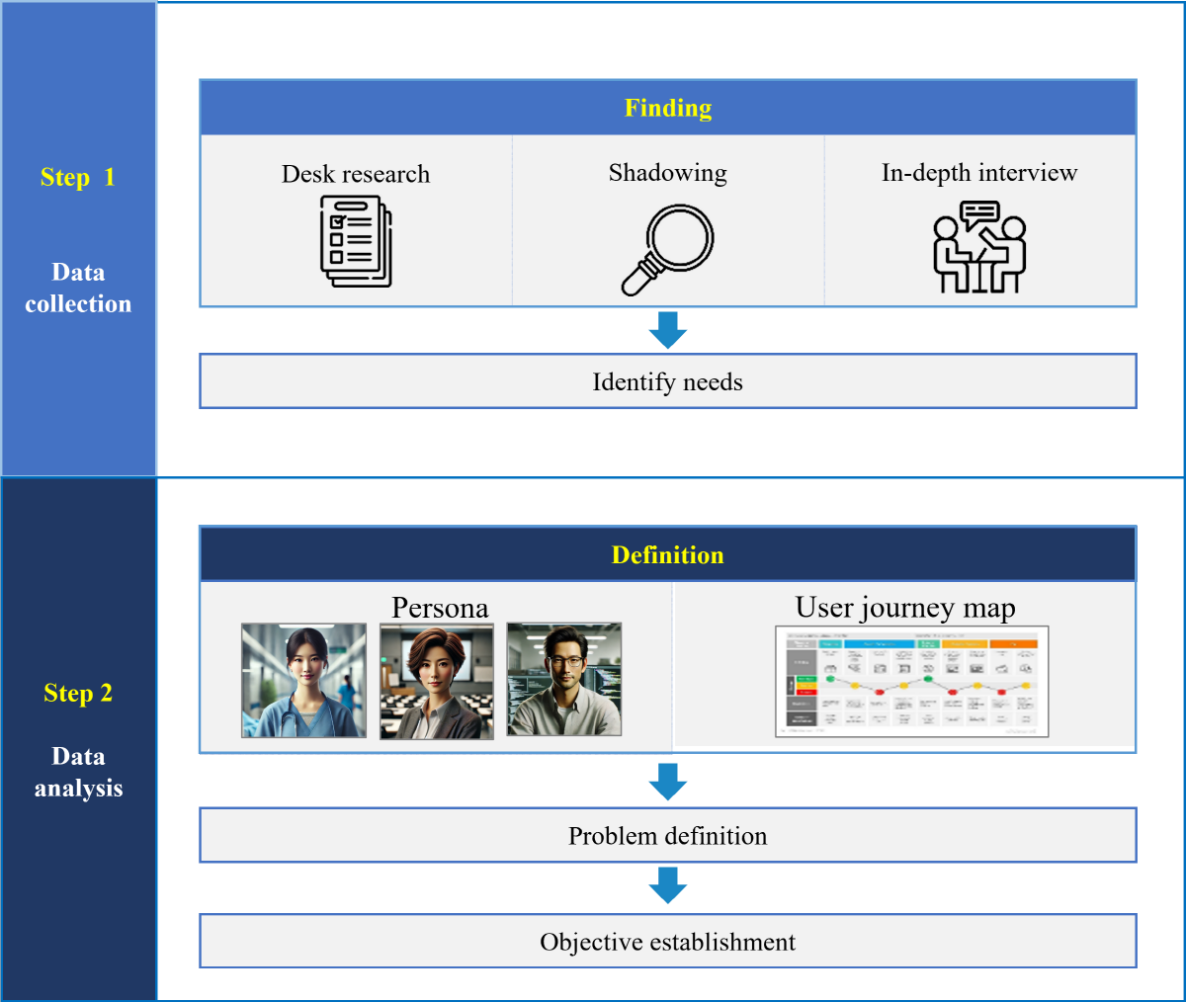
Research progress process: this figure outlines the research process, which comprises 2 stages: the first stage focuses on identifying users’ challenges and hidden needs, while the second stage involves defining the problem and setting service goals to address those issues.

This study consisted of 2 stages based on a review of the big data analysis and use procedure through desk research and a case study on the HBD platform (Figure 1). The first stage, discover, involves examining and understanding the problem situation to find the difficulties and hidden needs of those who use the service. Data research was conducted through shadowing and in-depth interviews with participants working on HBD analysis. The second stage, define, involves deriving major issues related to the problem based on analysis and contextual understanding of user survey results and setting service goals. Based on the needs of users explored through persona and user journey map, the AI agent’s design concept was proposed.

### Data Collection

Desk research examined prior documents and public institution announcements and papers searching for HBD and big data analysis. Platforms in overseas (PubMed and Google Scholar) and domestic (DBPia, Riss, and Kiss) databases were also searched. In-depth interviews took place over 3 weeks from February 1 to 21, 2023. The interview was conducted using a guideline consisting of 6 short-answer questions on the participant’s basics and open questions related to their HBD analysis and use journey. Based on the related literature [36] and expert experience, some main categories of difficulties and coping methods experienced in each stage of big data analysis arose, as well as desired support services used to solve the problem (Textbox 1). This was done after the initial formulation and underwent expert review.

**In-depth interview guideline: open-ended questions regarding users’ challenges and support needs at each stage of health big data analysis.**

**Collection**

- Talk about the type of data you used, and how to select and collect data.
- What was the most difficult part of the data collection process?
- How did you solve the difficulty?
- If you had a magic wand to solve any problems during the data collection phase, what would you solve?

**Preprocess**

- Talk about the process of preprocessing collected data (integration, conversion, and refinement).
- What were the difficulties you experienced during the preprocessing process and what was the most difficult thing?
- How did you solve the difficulties that occurred during the preprocessing stage?
- If you had a magic wand that could solve any problems during the preprocessing phase, what would you solve?

**Analyzing**

- Did you conduct the analysis yourself? If not, why?
- Was there any problem when you asked an expert to analyze it?
- What were the difficulties you experienced in the analysis stage, and what was the most difficult thing?
- How did you solve the difficulties that occurred?
- If you had a magic wand to solve any problems during the analysis phase, what would you solve?

**Visualizing**

- Was there any difficulty in interpreting and applying the derived results?
- What were the difficulties you experienced in the analysis stage, and what was the most difficult thing?
- What support do you need most during the result interpretation and visualization phase?

**Use**

- What features do you think should be included in artificial intelligence agents that support the use of health big data analysis?

To increase the realistic value of the data, the researcher visited the user’s studio where the computer was used by the participant for actual big data analysis and conducted one-on-one interviews. The individual interviews took about 1.5 hours. The process concluded when responses became repetitive, and no new information was generated. For aspects that were challenging to understand through verbal explanations alone, participants were asked to provide illustrative examples during the interview. With prior consent from the participants, the interviews were audio-recorded, and significant concepts identified during the discussions were documented in field notes. Each participant was interviewed once, and if further information was required during the data analysis phase, additional data were collected through follow-up phone calls or emails with the participants.

For 2 weeks, the researcher visited and shadowed 1 participant, a nurse researcher, who is currently conducting HBD analysis. While shadowing her in the laboratory, the researcher observed the participant’s behavior, and they captured the contact point of inconvenience and the moment when a problem occurred. To check for difficulties encountered by participants under different stages, from preprocessing to visualization of HBD, the researcher visited 3 times for a total of 1 hour per visit and interviewed the participant when important issues were found. Observed behaviors and environmental factors were recorded in field notes.

### Data Analysis

The interview data were transcribed by the researcher on the same day they were collected, and the transcriptions were analyzed at the levels of codes, subcategories, categories, and themes using qualitative content analysis procedures [37]. In the first stage, redundant content was excluded from the meaning units derived from the interviews, and codes were created to represent the core ideas using relevant concepts or phrases. In the second stage, the implicit meanings or content captured in the codes were abstracted to form subcategories. In the third stage, these subcategories were grouped into broader categories. Finally, in the fourth stage, themes were developed by integrating participants’ key experiences related to the HBD analysis process. To ensure the rigor of the analysis, the data were reviewed, analyzed, and discussed iteratively with the assistance of the second author (HC), a nursing professor with extensive experience in qualitative research. The findings were further validated for their universality and content validity through feedback from 2 research participants.

Based on insights confirmed through interviews and shadowing, personas and customer journey maps were designed to represent the participants’ experiences. A persona is a vivid and specific description of a potential end user and a fictional character representing the type of group to use the service; this is created by adding attributes to multiple subgroups identified within a potential user group [38]. Identifying users’ needs through personas formed by systematically classifying and embodying potential user groups can help present specific goals and directions for service development [39]. User journey maps are visualizations of the overall process and emotions of a persona experiencing the service over time; this helps in identifying and understanding the context of the problematic situation [30,40].

### Ethical Considerations

This study was approved by the institutional review board of the University of Keimyung (40525-202210-HR-059-03). All participants received a detailed information sheet about the research, were given the opportunity to ask questions, and were informed of their right to withdraw at any time without any negative consequences. Written informed consent was obtained from all participants, and to ensure confidentiality, all data were anonymized. To ensure confidentiality, all audio-recorded data were anonymized by removing personal information and assigning unique identification codes to distinguish participants. These data were securely stored on the researcher’s personal computer, equipped with security software, and were used solely for research purposes. Measures were taken to prevent any breach of personal information, violations of human rights, or ethical concerns, and the data will be permanently deleted after the study’s conclusion. Upon the completion of interviews and observations, a small honorarium was provided to the participants.

## Results

### Participants’ General Characteristics

Participants’ general characteristics are as follows (Table 1). Regarding the age of the participants, 6 (38%) were in their 40s. In terms of education, 6 (38%) held a master degree, and 10 (63%) held a PhD degree. As far as professions, professors (n=7, 44%) and researchers (n=4, 25%) accounted for the majority. Among the participants, 10 (62%) were health care personnel, and 6 (38%) were non–health care personnel. Regarding the experience of big data analysis, 4 (25%) were beginners, 6 (38%) had minor experience, and 6 (38%) had extensive experience. Formal data from public institutions were the most common type of data used (n=10, 62%). The analysis methods were statistical analysis (n=7, 44%), data mining (n=6, 38%), and text mining (n=5, 31%). Big data were used mostly for academic and research purposes (n=14, 88%).

**Table 1:** General characteristics of participants about age, occupation, education, and experience in health big data analysis (N=16).

Characteristics and categories		Values, n (%)
**Age (years)**		
	≤29	1 (6)
	30-39	4 (25)
	40-49	6 (38)
	50-59	4 (25)
	≥60	1 (6)
**Degree of education**		
	Master degree	6 (38)
	Doctoral degree	10 (62)
**Job**		
	Doctor	1 (6)
	Nurse	2 (12)
	Professor	7 (44)
	Researcher	4 (25)
	Company worker	2 (12)
**Experience of analysis**		
	Beginner	4 (25)
	Minority	6 (38)
	Majority	6 (37)
**Method of analysis^a^**		
	Statistical analysis	7 (44)
	Data mining	6 (38)
	Text mining	5(31)
**Type of used data^a^**		
	Structured data (public institution)	10 (62)
	Dielectric data	2 (13)
	Text data	5 (31)
**Purpose of use**		
	Scholarship or research	14 (88)
	Product or service development	2 (13)

^a^Multiple responses.

### Shadowing

As a result of observing the preprocessing of text data, which were unstructured data, the natural language processing stage had more procedures for manual verification than automatic tasks; this involved visually checking numerous data individually using analytical solutions. Researchers call this work manual labor due to the enormous amount of time spent on the work and considerable energy consumption. This pretreatment is an important task that can affect the outcome later if it is not completely refined at that stage. However, it has been found that nonanalysis professionals often deal with huge amounts of work without understanding the step-by-step progress, which often leads to situations wherein important parts are omitted.

Most users are trained on how to use the analytical solution before conducting big data analysis; however, they often forget the necessary commands or use because they do not have sufficient knowledge and are unfamiliar with the command line interface. In this case, the user wants to inquire about the information to an analysis-related expert, but there is no expert around to answer; hence, it was found that it was mainly solved by books or internet searches. However, this method took too much time, and in most cases, the desired information could not be found.

### In-Depth Interview

#### Overview

Based on the results of the in-depth interview, the user’s difficulties in the process of analyzing HBD showed a clear difference depending on the job. In the case of health care personnel, there was a slight difference in their experience when performing analysis work directly and requesting analysis work from analysts; yet, in both cases, there was no difficulty in understanding health data or finding potential meanings in analysis results and applying them to clinical situations. However, it was found that the major problems commonly experienced involve selection, installation, and use of analytical solutions and lack of knowledge and understanding of step-by-step analysis tasks that vary widely by data. Meanwhile, for non–health care personnel, difficulties in decision-making at various stages derived from a lack of health care knowledge have been identified as a major problem. These difficulties can be divided into 3: lack of knowledge on how to use analytical solutions (health care personnel), lack of crisis management ability (health care personnel), and lack of understanding of health care data and clinical decision-making ability (non–health care personnel).

#### Type 1: Lack of Knowledge on How to Use Analytical Solutions

In the case of health care personnel, beginners who did not have much experience in analyzing big data answered that they had difficulty in selecting and installing appropriate analytical solutions. Moreover, in the preprocessing and analysis stages, they did not know the package or library required for each step or did not know the syntax or function. One participant stated that:

I thought it would be over if I installed the analytical solution, but it was really painful to have to reinstall the library I needed for each task. I don’t know which package to use and how to install it, and how to enter the command after installing it. I don’t remember what I learned in the past, and it’s faster to do things manually, so most of the preprocessing were done manually.

Participant 4

#### Type 2: Lack of Crisis Management Skills

Participants responded that they had difficulty finding the cause and solution when an error occurred during big data analysis. In particular, if the error was not resolved, they could no longer proceed to the next step, and the whole process was completely interrupted; sometimes, even very simple problems that could be easily solved could not be found, so they spent days and days resolving them. One participant stated that:

I definitely put in the command as I learned, but I kept getting error messages. I don’t know what’s wrong, and I can’t ask anyone because there’s no one around me with this knowledge. I found Google and YouTubers to solve it, but it doesn’t work out. “Should I really give up here?” I had all sorts of thoughts. Later, through the expert, I found that the cause of the error was a mistake in the command. When I corrected it, it was executed in less than five minutes, but I was disappointed. I wasted three days without doing anything because of that and tried all sorts of ways by Googling.

Participant 7

#### Type 3: Lack of Understanding of Health Care Data and Clinical Decision-Making Ability

In the case of non–health care personnel, it was difficult for participants to determine the clinical significance or usability in the stage of selecting the analysis topic. In addition, for some domestic public data, participants found the data collection procedure too complicated. They responded that it was difficult to grasp the meaning of various variables included in the collected data due to a lack of understanding of health data and that it was cumbersome to merge data separated by year or variable. Regarding preprocessing, it was found that difficulties in preprocessing procedures such as normalization and missing value treatment, which changed the health care meaning and resulted in difficulty in decision-making, were greater than the difficulties related to the analysis itself. In addition, it was confirmed that it was difficult for participants to select variables to be used for analysis. One participant stated that:

To use data from the National Health Insurance Service, you have to submit IRB in the research plan, which is so complicated and difficult. The data collection stage was more difficult than the analysis. “Why did the industrial complex data divide the files into so many?” The examination data are difficult to combine because the items conducted every year are slightly different. There is an explanation file for variables, but there are too many variables in the data, so it is difficult to grasp. The test results do not clearly show the extent of the numerical value, so I can’t judge well. All the teachers who work in the hospital are busy, and there is no one close enough to approach personally and seek healthcare advice.

Participant 12

### Definition of Persona and User Journey Map

#### Overview

**Figure 2:**
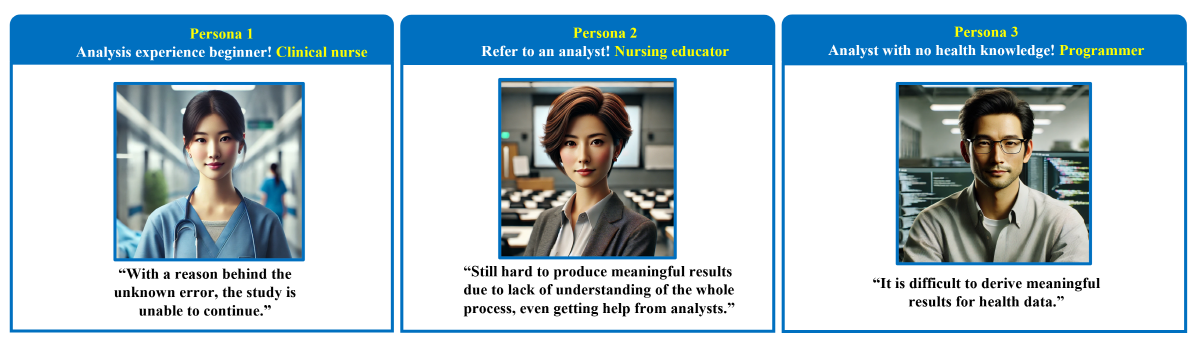
Three types of personas: persona 1: health care professionals as big data analysis beginners, persona 2: health care professionals who have experienced big data analytics, and persona 3: non–health care professionals who are experts in big data analytics.

In this study, when combining situations based on users’ actual experiences obtained through in-depth interviews, it was confirmed that analysis experience, occupation, and direct performance of analysis work act as the main factors affecting the user’s experience. Accordingly, 3 personas were set up: “health care professionals as big data analysis beginners,” “health care professionals who have experience in big data analytics,” and “non–health care professionals who are experts in big data analytics.” Based on this, needs and pain points from the user’s perspective were identified (Figure 2).

#### Persona 1: Health care professionals as big data analysis beginners

**Figure 3:**
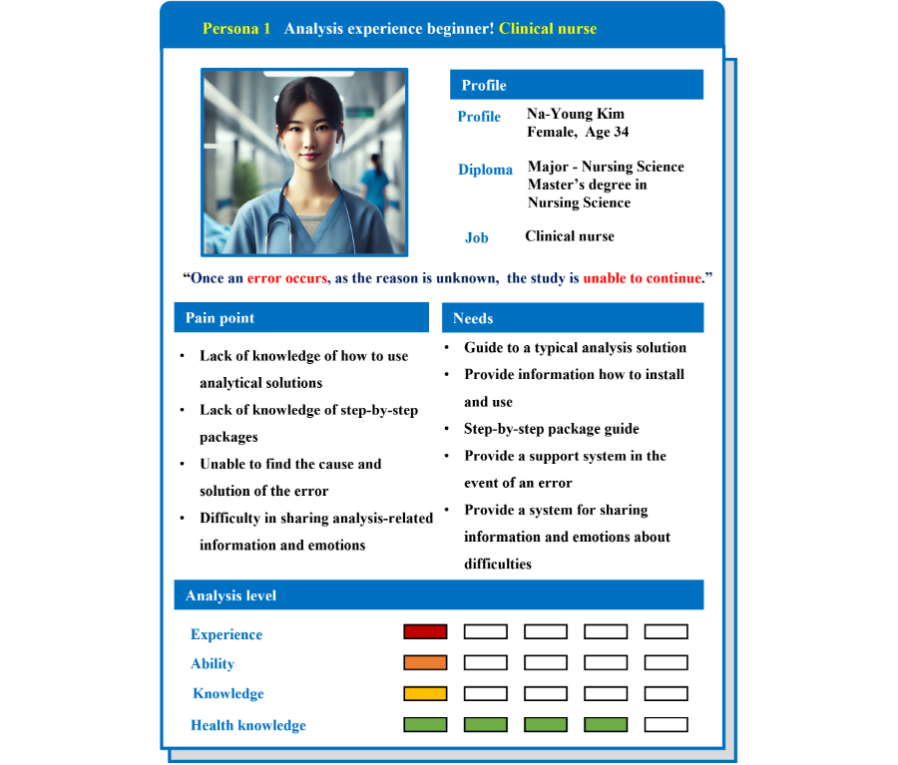
Persona 1: health care professionals as big data analysis beginners: represents novice medical personnel with limited experience in big data analysis. This persona lacks knowledge of analysis tools and solutions and has minimal experience using them.

Persona 1 is a persona that represents a health care practitioner with entry-level analytical experience (Figure 3). They lack knowledge of analytical solutions, experience frequent errors in progress due to unfamiliar use, and have difficulty finding solutions. Therefore, they need a support system that can provide information on how to use the analytical solutions and query errors or to be able to ask questions real time during the progress stage.

**Figure 4:**
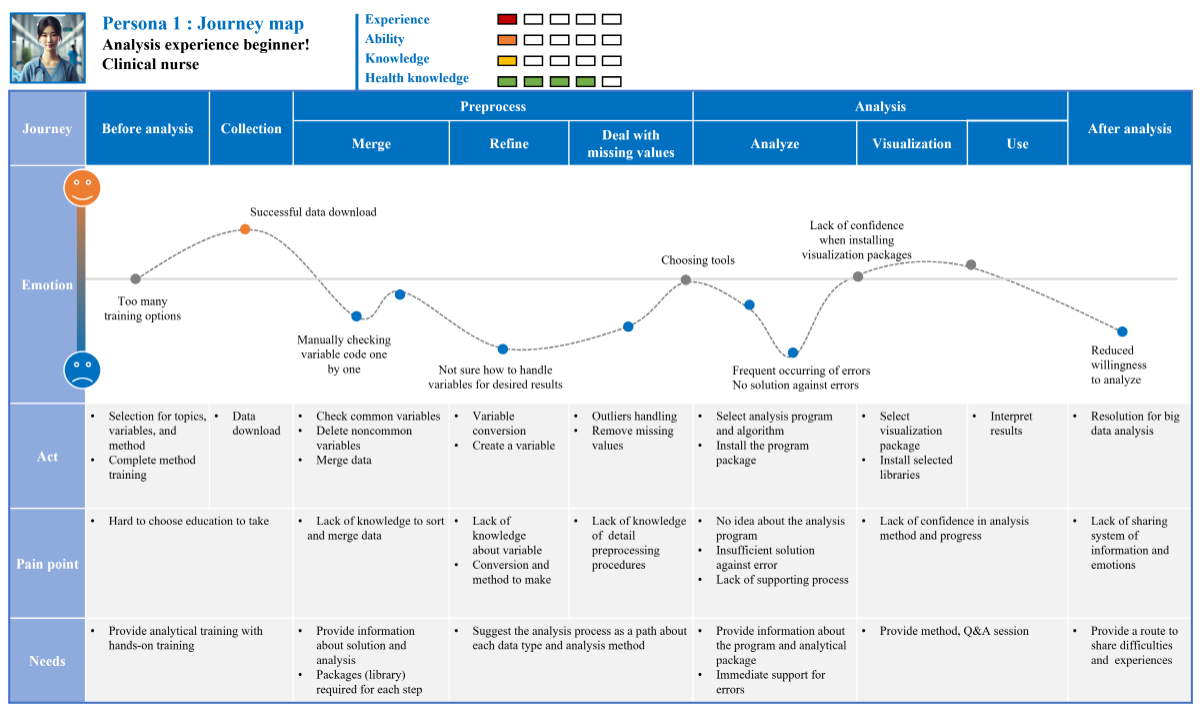
User journey map of persona 1: highlights frequent errors occurring during the data preprocessing and analysis stages. The inability to identify the causes of these errors leads to significant time consumption and frustration. Q&A: question and answer.

The user journey map prepared based on this persona is shown in Figure 4. “Na-Young Kim,” who works as a clinical nurse at a university hospital, recently conducted a potential class analysis study on “risk factors for metabolic syndrome in single-person households.” Since she has never performed big data analysis before, she completed analysis-related training before conducting the research. She was unfamiliar with how to use the analytical solution in the preprocessing process, so she had to proceed with most of it manually, which consumes a lot of time and energy. In the complete analysis stage, an error occurred during the command input process, but the progress was stopped because the cause of the error could not be found. She searched for information through Google and tried to modify it by referring to the provided notice, but it was not solved, so she spent days and days working on the problem. The cause of the error, which was confirmed through experts who were barely connected after many twists and turns, was unexpectedly too simple, and the problem was solved in 5 minutes. She thinks several times that it would be nice to have someone who she can ask for advice on something. After many twists and turns, the analysis was completed, but with the idea that such a difficult experience is enough once in a lifetime, she decided that she will never do big data analysis research again.

Which part of it is wrong? I don’t know, there’s no one I can ask, and once an error occurs like this, everything will be completely stopped, so it’s really hopeless. I can’t believe I held on to a problem that would be solved in five minutes for four days. It’s so frustrating.

#### Persona 2: Health Care Professionals Who Have Experienced Big Data Analytics

**Figure 5:**
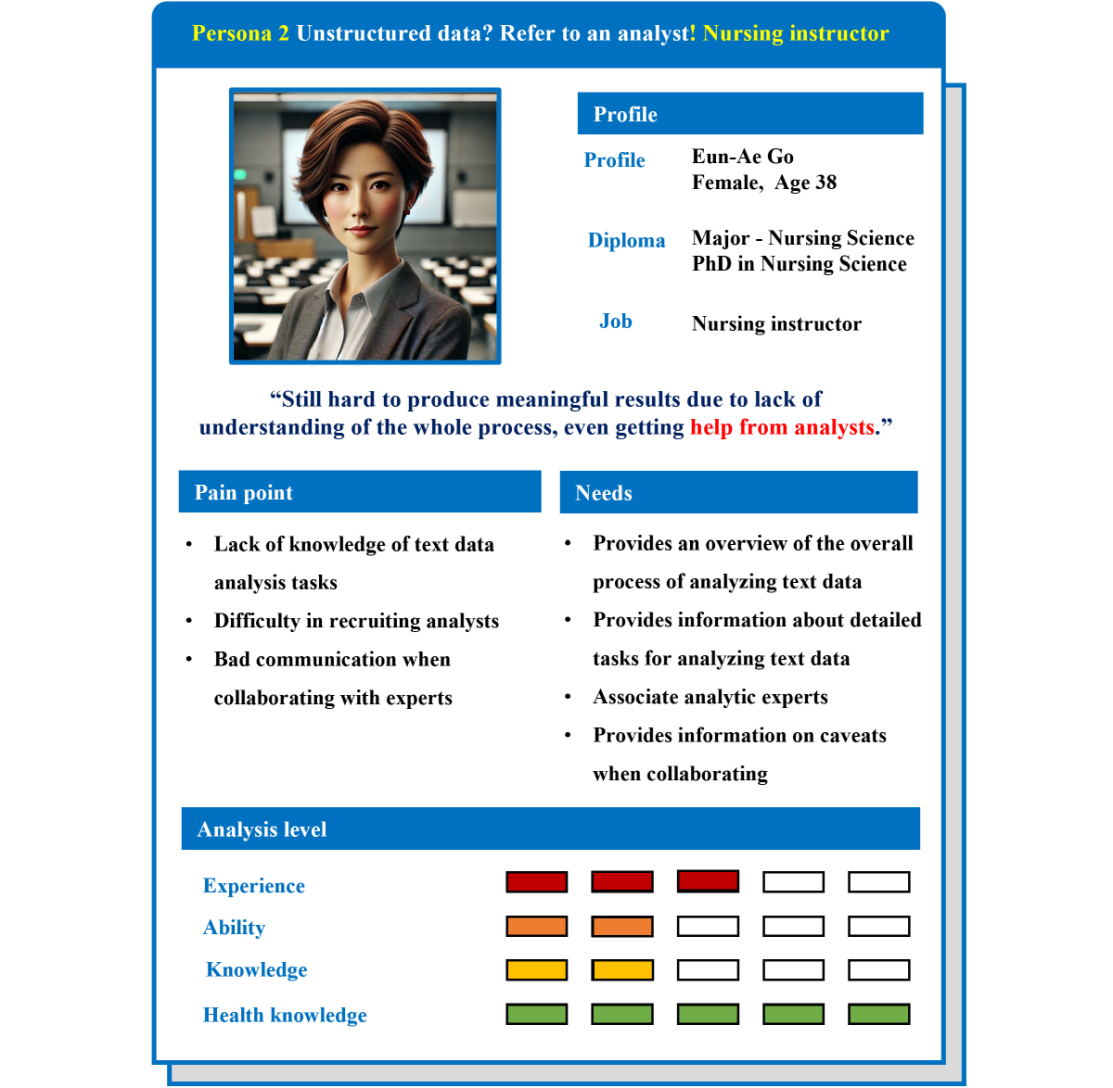
Persona 2: health care professionals who have experience in big data analytics: represents medical personnel who outsource big data analysis tasks to experts. This persona lacks an understanding of the overall analysis process and methods.

Persona 2 is a persona that represents the experience of a health care professional who commissioned an analysis expert to perform the analysis (Figure 5). In this case, if the client lacks an understanding of the overall process of big data analysis or does not communicate sufficiently in advance, disagreement may occur during the process. Therefore, it was found that users who conduct big data analysis through collaboration with analysts need a system that provides information on the contents and precautions to be discussed in advance when requesting analysis, guides the entire process of big data analysis, and provides explanations.

**Figure 6:**
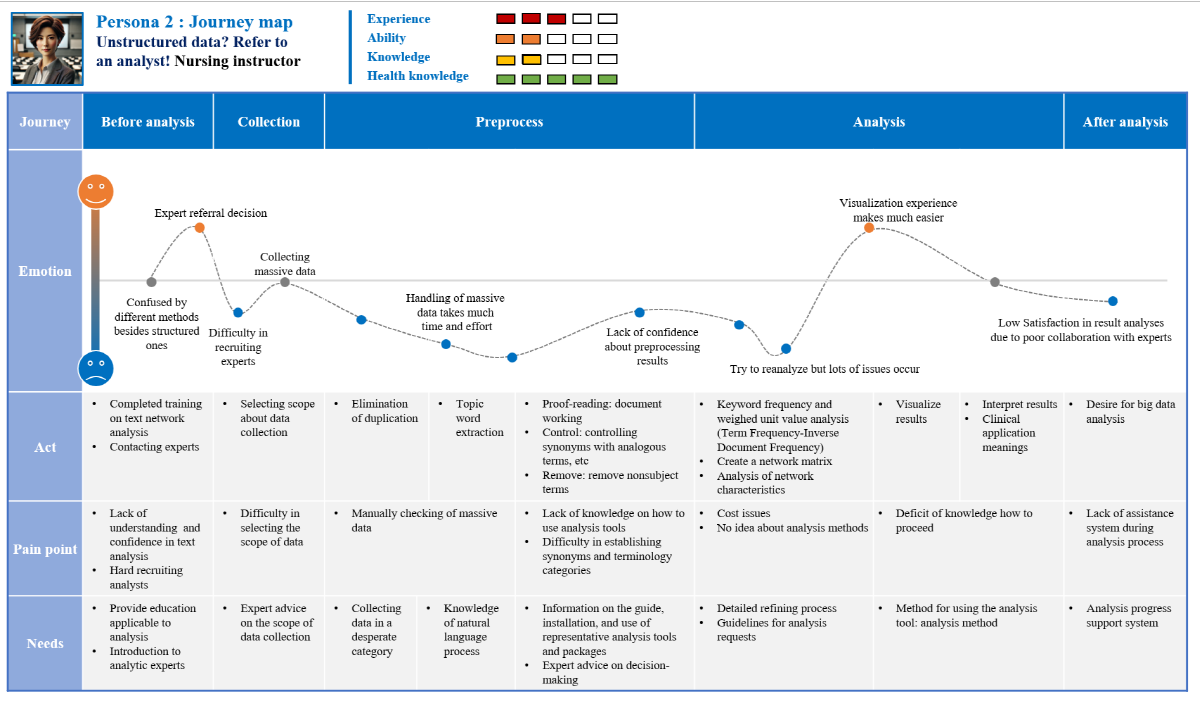
User journey map of persona 2: illustrates difficulties in communication and disagreements during the collaboration process, stemming from limited knowledge of the analysis process and methods. These issues result in constraints on the quality and scope of the analysis outcomes.

The itinerary map for persona 2 is shown in Figure 6. “Eun-Ae Go” is a lecturer who is currently teaching students at a nursing university and recently conducted a study to identify “severe COVID-19 risk factors” using HBD. Although she has previously conducted several studies using HBD, it was her first time analyzing a network using text data; hence, she completed several training sessions on using network analytical solutions before starting the analysis. She did not have an expert in big data analysis around her, and she could not make a decision because she did not trust the companies she found on the internet. She finally got in touch with an engineering major through a few acquaintances and asked them to do both data preprocessing and analysis. It was found after the end of the analysis that the resulting values were affected by texts that were not completely refined during the preprocessing process. She wanted to reanalyze by repurifying, but the analyst did not agree to the reanalysis due to time and cost issues. Through this, she realized that sufficient communication and consultation must precede when requesting analysis work from others; there is also a limit to discovering and interpreting the meaning of the data only with the analysis results conducted by the AI agent without understanding the analysis process or method. Next time, she intends to perform the pretreatment and analysis herself, even if she is not an expert.

You need to know something when you ask others to do it. Even if we ask experts to analyze big data, we can produce meaningful results only when we have a basic understanding and knowledge of the overall analysis process and method.

#### Persona 3: Non–Health Care Professionals Who Are Experts in Big Data Analytics

**Figure 7:**
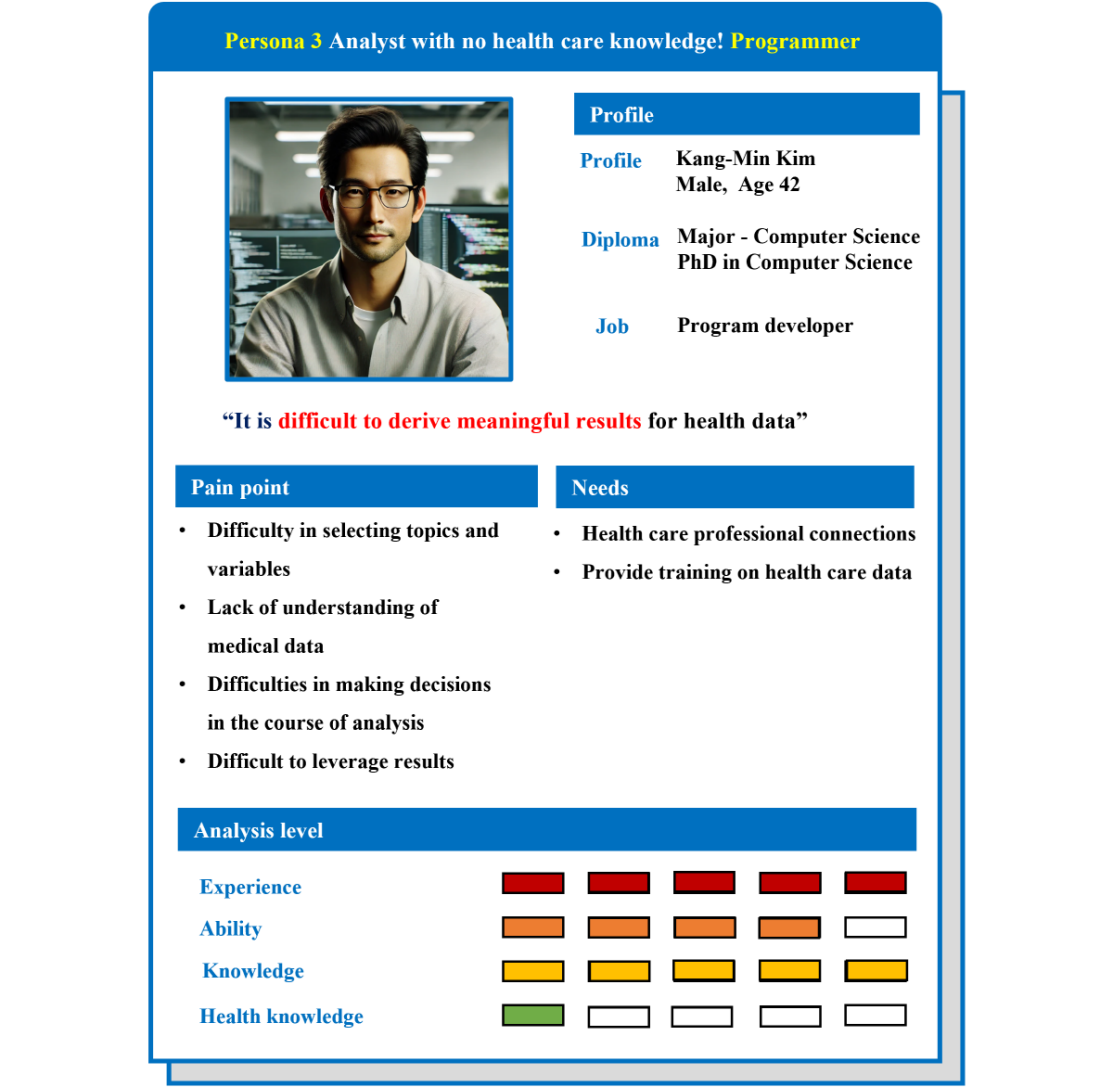
Persona 3: non–health care professionals who are experts in big data analytics: represents a nonmedical professional proficient in big data analysis but lacking expertise in health care. This persona struggles with understanding health data and clinical knowledge.

Persona 3 is a persona representing people who are experts in big data analysis but have no knowledge of health care (Figure 7). They are good at big data and analysis, but they experience continuous doubts and confusion during the analysis process due to a lack of health care knowledge. Through this, it was found that non–health care users who are fluent in analysis need a system that provides information on health care data. They also need to seek advice from health care experts in decision-making situations that occur during the analysis process.

**Figure 8:**
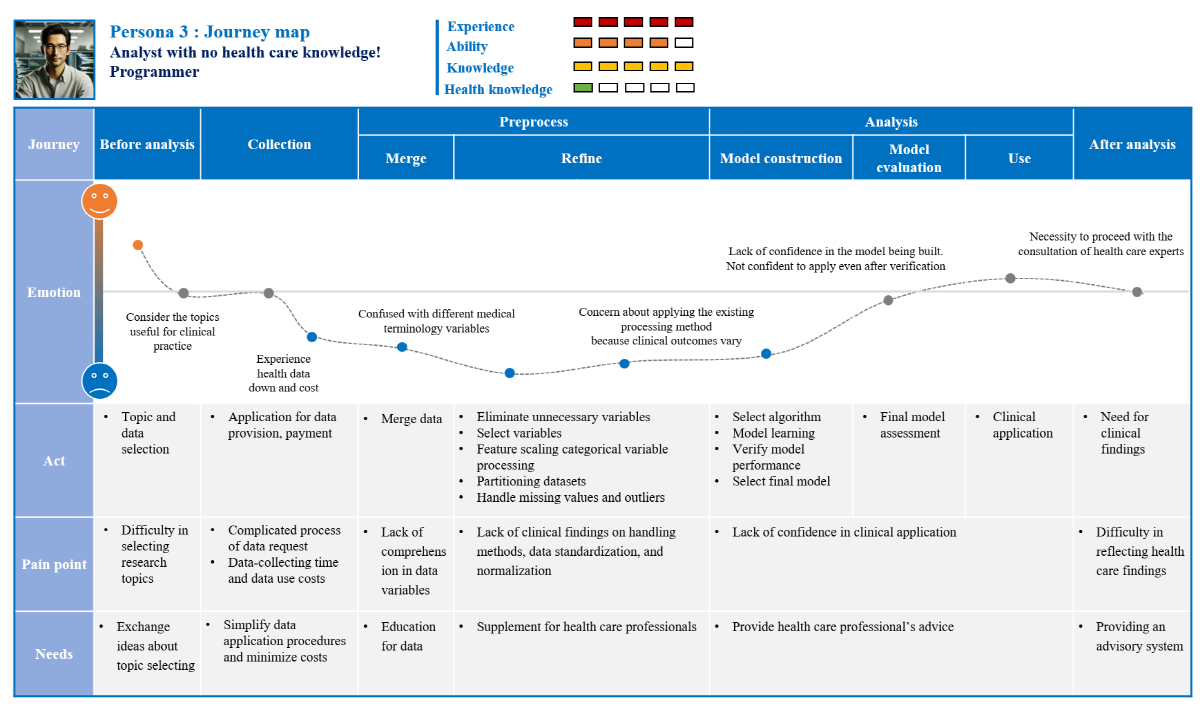
User journey map of persona 3: describes experiences of confusion and uncertainty when situations requiring clinical judgment and decision-making arise at various stages, including preprocessing, analysis, and postanalysis.

The itinerary map for persona 3 is shown in Figure 8. Currently working as a programmer, “Kang-Min Kim” is a computer science major and a big data analysis expert. Several studies related to big data analysis in various fields have been conducted. Recently, interest in HBD with infinite use value has increased, and research on “building a diabetes prediction model” has been conducted using the National Health Insurance Service’s screening data. Since “Kang-Min Kim” is good at analyzing big data, he predicted that there will be no special variables or difficulties in analyzing big data in the process of HBD analysis. However, unlike previous data analysis work, he felt uncertainty and doubt throughout the analysis process due to the nature of the health data that the analysis findings of the machine were not 100% reliable. Due to a lack of understanding of the meaning of each variable in the health data, the impact of normalization and missing value processing methods on future analysis results, the selection of input variables when applying machine learning, and clinical meaning and application of derived results, among others, he had doubts that he could not be sure of the results of the work that he decided and implemented alone without communication with related experts. At the end of the work, it was deduced that clinical judgment and opinions of health care experts must be combined in the analysis process to properly use HBD. In the future, he intends to conduct HBD analysis through collaboration with health care experts.

This numerical value is an abnormal range, how much does this abnormal mean? Does this process affect the clinical meaning of data values? I can’t judge because I don’t have healthcare knowledge. A proper analysis of health big data requires not only being good at analysis itself.

### Deduction of Needs

The user’s needs derived for AI agent design that supports the use of big data analysis are as follows.

First, there is a need for a service that provides a guide for analytical solutions, which can be easily used by the public and provides information on how to use the solution to proceed with each step. For nonanalysis professionals, the knowledge of the various libraries (packages) and execution methods required for several tasks to be performed at each stage of preprocessing, analysis, and visualization was unclear; even those with experience did not know how to use the solution. Therefore, users will be able to conduct big data analysis more easily if they have a guide for analytical solutions that are easily available to the public and provide detailed explanations on how to use the solutions for step-by-step tasks according to the progress procedures for each data type.

Second, there is a need for a service that guides the overall process of big data analysis in detail. Big data analysis takes place through 4 stages: data collection, preprocessing, analysis, and visualization and use. Most of the big data analysis platforms in other fields currently operate these 4 stages and provide services such as data introduction and transaction services, data analysis tools introduction and installation guidance, analysis-related training guidance, data analysis and use case sharing. However, there is no system that guides detailed work procedures and specific methods required for each stage. Ordinary people, who are not big data analysis experts, need a service that guides them in their overall analysis journey and provides detailed explanations of the specific tasks to be performed at each stage, just like with navigation, wherein a person arrives at the destination if guided.

Third, there is a need for a system where one can ask or seek real-time advice from analysis experts about problem situations or questions that occur during analysis work. Users may encounter problem situations in the analysis process, and there may be questions that they want to be confirmed by experts. These problems should be dealt with quickly because if they are not solved, they cannot proceed to the next step. However, it is not easy for nonexperts in the field to find the cause of the problem, resulting in a significant waste of time.

Fourth, there is a need for a service that introduces and links analysis experts who can request analysis work or health care experts who can provide health care advice. There may be situations wherein health care personnel need to seek advice on big data analysis and non–health care personnel need to seek health care advice, but it is not easy for users to find these people on their own. It is necessary to provide a channel to meet necessary professionals and at the same time provide guidance on the contents of prior consultations or precautions necessary for collaboration.

Based on the earlier-mentioned problems and user needs, we present five design concepts: (1) personalized platform for the user level, (2) appropriate direction through a navigation function, (3) crisis management support system through question and answer, (4) communication and sharing opportunities between users, and (5) expert-linked services. In addition, it is believed that these concepts will be able to meet users’ needs by adding experience or information related to HBD analysis, providing guidance on health data introduction and access procedures, giving useful big data–related education introduction, and having one-on-one consultations.

## Discussion

### Principal Findings

To design appropriate and useful technologies or services, it is important to focus on the applicable user’s experience and consider the user’s individual needs [41]. Recently, this user-centered approach has been actively used in health care–related service development, such as in studies on the design of eHealth AI services [42], smart home system design [43], and web-based agents to support physical and mental health self-management in patients with chronic obstructive pulmonary disease [44]. In this study, before developing an AI agent that supports the use of HBD analysis, the experience and needs of users who have performed big data analysis by applying a human-centered design methodology were identified. The discussions on the 5 service concepts derived through this and the goals and contents to be achieved through each service are as follows.

First, the service is a personalized platform that fits the level of the first user. As a result of the study, the demand for a system that supports big data analysis was different for each user type. The analytical solution of the analysis environment provided in the existing big data platform focuses only on creating an analytic environment and building necessary analytical solutions rather than meeting the needs of users [45,46]. These platforms are not a problem for analysts to use, but in the case of ordinary users, they do not even know how to use a given analytical solution, so the platform created is practically irrelevant or meaningless. Therefore, it is important to provide an analytical environment that fits the user’s level and includes detailed information required by the user [47].

In a study on web-based platform development to support participants and caregivers with memory problems, it was also found that the evaluation of the usefulness of the function was different for each group due to different priorities for platform functions between user groups. As a result, a customized platform was designed to meet the needs of a specific group [48]. Guiding cost-free, easily available analytical solutions, and providing necessary information according to the user’s needs such as installation methods, necessary libraries, and commands, will reduce the fear of using analytical solutions by inexperienced users. This is supported by examples of successful results in weight, blood pressure, and blood sugar management by characterizing users in the development of a meal planning platform that supports healthy eating development and providing services such as customized nutrition assessment for each user, personalization of meal plans, and changing the environment of individual food purchases [49].

Second, the service provides an appropriate direction presentation system through a navigation function. Most health care professional users were found to lack knowledge on detailed big data analysis processes that vary by data type. If a detailed guide is provided for the analysis progress procedure for each type of representative data, like a road guide navigation, even inexperienced beginners can follow the guide and conduct the analysis on their own. In addition, this can improve understanding of the overall analysis process. In a digital platform designed for youth mental health services, the path of each service needs to be presented, and the content of the service provided by each route is different so that users map along the treatment path determined by case and are guided by the platform to continuously implement it according to the process [50].

Third, the service is a crisis management ability support system through question and answer. A service that allows users to raise queries and answer questions or errors that arise during analysis operations helps users solve problems quickly and reduces unnecessary time and energy waste. A platform development study, which supported operators who manage big data information processing systems, was also equipped with an intelligent web-based system based on AI that helps system operators cope with problems that occur occasionally and obtain the necessary information [51]. In the development of a mobile platform to support patient participation and reporting on lung cancer surgery, a channel was provided to ask patients using the program about questions or concerns that may arise during and after surgery or to report various suspected exacerbations. This allows users to hear immediate health care team explanations, promoting their ability to solve problems [52].

Fourth, the service creates a venue for communication and sharing that provides opportunities for exchange between users. There is a need for a channel of communication that can exchange experiences or information related to big data analysis and share effective big data use cases. Through this, the user may obtain important information and achieve new collaboration through communication. PatientsLikeMe (PatientsLikeMe Inc), a platform that provides information on rare diseases, provides a channel for users to share various information such as their health status and symptoms, drug effects, and side effects. It is not only useful to other users, but it has also resulted in an increase in the number of subscribers to the platform and further expansion of the platform’s capabilities, with accumulated data being sold back to necessary institutions [5,6]. The survey on user needs for digital mental health platforms also revealed that the community was a major topic. It has been found that users do not hide their information about diseases or treatments but rather want to connect and share experiences with others, thereby developing the platform’s capabilities and believing that new use cases can be developed [53]. In a digital platform designed to enhance cancer treatment support for adult survivors of cancer, survivors of cancer were also found to receive psychological support by expressing various interests such as eating habits and mental health to peer and patient support groups and sharing experiences [5,8].

Finally, the service provides a connection service with experts. Users who want to request analysis work, analysts, or those who need advice from health care experts are required to provide services that link related experts, and these services enable active use of big data through collaboration. PatientsLikeMe also uses services that link institutions that run clinical trials on new drug development with patients who want the opportunity to use a drug being tested.

### Strengths and Limitations

This study adopted a human-centered design approach to enhance the usability of HBD, successfully identifying the unique needs and challenges faced by various user groups. Through in-depth interviews and shadowing techniques, we gained significant insights into the emotional and cognitive aspects of users’ experiences during the analysis process. The development of 3 distinct personas and journey maps revealed that each user group has specific requirements. For instance, novice health care professionals require more guidance and support during critical situations, whereas experienced data analysts prefer advanced features and customized settings tailored to various data types. These findings indicate that services provided by the system cannot be uniform and underscore the necessity for personalized user support. Additionally, while many users seek expert advice or communication with peers who have similar experiences, there is a notable lack of accessible communities dedicated to health data analysis and use. This highlights the need for developing user-friendly systems capable of offering practical support to users in the future. Our study has several limitations. First, the in-depth interview participants were primarily users who analyze and use widely used health data, which may limit the generalizability of the findings to users working with less common or emerging types of health data. As new types of health data continue to emerge, the specific demands and analytical experiences associated with these data have not been fully incorporated into the current personas and user journey maps. Additionally, this study may not be generalizable to users outside of the South Korean environment, as the findings are context-specific.

When the number of participants is expanded in future research, the personas and journey maps developed in this study may require reorganization, modification, or expansion beyond the 3 types presented. Like all in-depth studies, the results of this research cannot be standardized but aim to broadly capture the complex and challenging experiences of users regarding HBD analysis and use. Nevertheless, the personas and journey maps created in this study can serve as a foundational guide, gradually expanding and adapting to meet the needs of a wider range of users and data environments. Future research should conduct in-depth demand analyses targeting users of emerging data types to more comprehensively reflect diverse user experiences.

### Conclusions

The ultimate purpose of precision health care is to provide optimal treatment and treatment tailored to consider individual genetic, environmental, and lifestyle factors based on health care information. This can be realized through an effective use of big data. This study proposes a human-centered design to develop an AI agent supporting the use of HBD to increase use. Based on the needs of users through shadowing and in-depth interviews, 5 key implications for HBD analysis and use support system (personal platform, navigation function, crisis management support, communication and sharing, and experts) were revealed.

In the future, we propose a follow-up study to develop a system that supports the overall process of big data analysis based on the implications derived through this study. This is expected to further increase big data use by helping many users who hesitate to use big data due to difficulties in the analysis process to analyze big data more easily.

## Abbreviations

AI: artificial intelligence
HBD: health big data

## Data Availability

The datasets generated and analyzed during this study are not publicly available due to privacy restrictions but are available from the corresponding author on reasonable request.
